# Review of neuroprotective potential of natural products against hypoxia-induced neuronal injury

**DOI:** 10.3389/fphar.2026.1746683

**Published:** 2026-02-11

**Authors:** Yuman Zhang, Xingyu Yi, Dingzhong Wang, Cunlong Kong, Yan Xu, Jianping Xie

**Affiliations:** 1 Key Laboratory of Tobacco Flavor and Fragrance Basic Research of CNTC, Zhengzhou Tobacco Research Institute, Zhengzhou, China; 2 School of Basic Medical Sciences, College of Medicine, Zhengzhou University, Zhengzhou, China; 3 Beijing Life Science Academy (BLSA), Beijing, China; 4 Department of Pain Management, The First Affiliated Hospital of Zhengzhou University, Zhengzhou, China

**Keywords:** cerebral ischemic diseases, hypoxia/ischemia, ischemic stroke, natural ingredient, neurodegenerative disease, neuroprotection

## Abstract

Neurological disorders such as neurodegenerative diseases (NDDs) and stroke have become a major global health burden. Evidences from several studies suggest that their pathogenesis is related to hypoxia. However, there are certain limitations and adverse effects associated with the current treatments for neurological disorders. Studies have shown that some natural products and their extracts—such as (−)-epigallocatechin-3-gallate, *Centella asiatica*, ginkgolides, quercetin, berberine, and curcumin, which are the focus of this paper, along with briefly mentioned resveratrol and compounded preparations—have some neuroprotective effects in hypoxia-induced neurological injury. Owing to their favorable safety profile and minimal adverse effects, they have attracted widespread attention. Moreover, their primary mechanisms of action possibly stem from oxidative stress inhibition, neuroinflammation attenuation, and neuronal apoptosis reduction, providing potential approaches for the prevention and treatment of neurological diseases. In this review, we searched the PubMed and Web of Science databases for relevant literature collected over the past 35 years. Overall, we summarized the neuroprotective effects of these natural products against hypoxia-related neurological injury, focusing on the molecular mechanisms and signaling pathways, thereby offering a theoretical basis for further research on the specific neuroprotective mechanisms and drug targets of their observed preventive and therapeutic effects on NDDs, primarily Alzheimer’s disease (AD), Parkinson’s disease (PD), and Huntington’s disease (HD) in this review.

## 1 Introduction

Oxygen (O_2_) is essential in aerobic respiration and metabolism. In eukaryotic mitochondria, O_2_ acts as the final electron acceptor in the electron transport chain to produce ATP for energy supply (Taylor, 2008). Consequently, when tissue O_2_ levels decline and O_2_ demand exceeds supply, cells are under-supplied, and their normal physiological functions are compromised. The brain, as a high-energy-demanding organ, is unusually sensitive to hypoxia (Bailey, 2019). Thus, transient interruptions in O_2_ supply might trigger neuronal death, neuroinflammatory cascade responses, and blood-brain barrier (BBB) disruption, ultimately leading to cognitive impairment or irreversible neurological deficits. The incidence of neurological disorders associated with hypoxia (including neurodegenerative diseases [NDDs] and ischemic strokes) increases yearly, alongside the global percentage of disability due to strokes and NDDs, which has also continued to increase over the past decade (Global burden of disease study 2019, available from: https://vizhub.healthdata.org/gbd-compare/), making neurological disorder a major global health challenge.

NDDs are a group of lesions that affect the central and peripheral nervous systems, resulting in progressive loss of neuronal structure during neurodegeneration, culminating in neuronal cell death and loss of function (Xiang et al., 2024). We mainly discussed NDDs including AD, PD, and HD in this review. The exact pathophysiological mechanisms of NDDs remain elusive due to the complexity of their causative factors. This paucity of data complicates drug design aimed at targeting specific molecules or pathways (Chen et al., 2025a). Notably, many NDDs, such as AD and PD, have similar underlying mechanisms, with numerous cases linked to O_2_ imbalance (Rey et al., 2023). In several NDDs, the pathological process is often characterized by hypoxia, especially in AD (Wang et al., 2021a; Peers et al., 2009). Regarding hypoxia-based treatments for NDDs, several preclinical and clinical studies have demonstrated that hyperbaric oxygen therapy (HBOT) significantly improves symptoms in models and patients with NDDs as well as in those with other neurological disorders such as stroke and traumatic brain injury (TBI) (Mensah-Kane and Sumien, 2023). However, increased O_2_ might lead to excessive oxidative stress damage (Thom, 2009); moreover, there are adverse effects associated with HBOT, including rare and serious complications. In addition, studies have not determined if the therapeutic effects of HBOT are due to its direct effects on brain function or an indirect one by enhancing medication efficacy (Somaa, 2021). In summary, despite continued research, no cure has been discovered for any of the NDDs, underscoring a significant challenge in this area of research (Heemels, 2016).

Approximately 25% of the global population will experience a stroke in their lifetime. Annually, >12 million people experience stroke, and 7.3 million of those individuals die as a result (http://world-stroke.org). Basically, stroke can be categorized as ischemic or hemorrhagic. This review will concentrate on the treatment of ischemic stroke associated with hypoxia. Acute ischemic stroke (AIS) is a medical emergency that occurs when blood flow to a part of the brain stops suddenly, resulting in brain cell damage and loss of neurological function (Walter, 2022). Currently, the main clinical treatments for AIS are intravenous thrombolysis and mechanical thrombectomy (MT) (Walter, 2022). The primary limitations of this approach include a limited time window and the occurrence of adverse effects, such as bleeding. Moreover, the residual neurological deficits observed in most survivors underscore the urgent need for the development of adjuvant neuroprotective agents. Consequently, natural products, which have multiple mechanisms and drug targets, have emerged as promising therapeutic candidates. Furthermore, a notable advantage of natural products is their reduced potential for adverse effects, which is a major advantage in pharmaceuticals. Recently, natural products such as (−)-epigallocatechin-3-gallate (EGCG), *Centella asiatica* (CA), ginkgolides, quercetin (Que), berberine (BBR), and curcumin have demonstrated anti-hypoxic neurological injury effects in animal and cellular models by modulating hypoxia-inducible factor 1 alpha (HIF-1α), inhibiting oxidative stress, attenuating neuroinflammation, and decreasing apoptosis, among other activities. This study aimed to review and summarize available research on the ameliorative effects of six natural products with potential neuroprotective properties against cerebral ischemia-hypoxia injury, and further highlight the direction of future research on the specific neuroprotective mechanisms and drug targets of the observed preventive and therapeutic effects of these natural products. We also sought to provide a theoretical basis for the prevention and treatment of related neurological disorders.

## 2 Search strategy

In this review, we searched the PubMed and Web of Science databases for English articles published between 1991 and 2025 using keywords such as “natural products,” “hypoxia,” “neurodegenerative diseases,” “ischemic stroke,” and “neuroprotection.” After reviewing the literature and excluding studies with content not closely related to our topic, as well as studies of inferior quality, we finally included 233 full-text articles in the review after summarizing and synthesizing them. Ultimately, we selected six natural products—EGCG, CA, ginkgolides, Que, BBR, and curcumin—for detailed elaboration due to their extensive research and closer association with hypoxia protection. The remaining compounds will be briefly discussed. We reviewed articles that were directly related to hypoxia, natural products, and NDDs. In the process of evaluating the articles, we further searched and explored the references of high-quality articles and reviews and added them to this review.

## 3 Natural products

### 3.1 EGCG

Green tea (*Camellia sinensis*), the first tea to be discovered in the world, originated in China and has since spread globally. Aside from water, green tea is recognized as the most popular beverage consumed worldwide (Zhao et al., 2022). Moreover, green tea is a type of unfermented tea, which prevents the oxidation of polyphenolic components and largely preserves the natural components of fresh tea leaves, especially catechins. Catechins are colorless, astringent, water-soluble compounds; the main essential components of green tea (Graham, 1992). EGCG is the most active of the six major catechin compounds contained in green tea (catechin, gallocatechin, epicatechin, epigallocatechin, epigallocatechin gallate, and EGCG), accounting for 10%–15% of the total catechins (Ohishi et al., 2016) and also the most abundant and active compound in green tea (Zhao et al., 2020). Emerging evidence suggests that EGCG possesses diverse pharmacological activities, including anti-inflammatory (Ohishi et al., 2016; Tasneem et al., 2019), antioxidant (Karas et al., 2017; Guo et al., 2005), anticancer (Baláži et al., 2019), anti-apoptotic (Guo et al., 2005; Weinreb et al., 2004), and neuroprotective (Cheng et al., 2021; Payne et al., 2022) properties, highlighting its therapeutic potential for the treatment of various human diseases (Chakrawarti et al., 2016). EGCG is approved as a dietary supplement in many regions worldwide, including Europe, the United States, Canada, and Japan. However, doses exceeding 600 mg per day may cause liver toxicity (Dekant et al., 2017). Several studies have shown that EGCG exerts important neuroprotective effects against a variety of neurological diseases/disorders such as AD, PD, cerebral edema, and cerebral ischemic injury (Xu et al., 2016; Zhang et al., 2020a; Park et al., 2020). However, due to poor physicochemical stability and low oral bioavailability of EGCG, its clinical application remains limited. Research indicates that certain technologies, such as nanotechnology, can enhance its stability, efficacy, and pharmacokinetics by encapsulating EGCG within nanoparticles (Peng et al., 2025).

Moreover, reports suggest that HIF-1 facilitates endogenous neuroprotection under hypoxic/ischemic conditions. HIF-1 is a heterodimer (including HIF-1a and HIF-1b), with the HIF-1b subunit constitutively expressed and the HIF-1a subunit regulated in an O_2_-dependent manner. HIF-1α—the active subunit in HIF-1—is a key regulator of cellular and systemic O_2_ homeostasis, and it regulates the expression of multiple genes (Lu and Kang, 2010; Lee et al., 2020). The study by Orly Weinreb et al. systematically revealed the neuroprotective mechanism of EGCG in a human neuroblastoma SH-SY5Y cell model induced by prolonged serum deprivation. Gene expression analysis was utilized to ascertain EGCG effects on protein levels and mRNA expression of the prolyl 4-hydroxylase subunit. The findings demonstrated a reduction in both protein levels and mRNA expression following EGCG treatment. Notably, this enzyme, which belongs to the HIF prolyl hydroxylase family, serves as a key iron oxide sensor for hypoxia perception and can also negatively regulate the stability of multiple enzymes and the degradation of proteins involved in cell survival and differentiation. Subsequent studies have indicated that EGCG may stabilize HIF-1α through various regulatory pathways, including immunoglobulin-heavy-chain binding protein HSP 70 kDa protein 5 (BiP), prolyl 4-hydroxylase, HSP90 beta, and E2 ubiquitin-conjugating enzyme, among others. This mode of regulation corroborates with proteomic data. According to proteomic analyses, EGCG increases the levels of cytoskeleton structural proteins and binding protein 14-3-3 γ, and is involved in regulating intracellular signaling pathways as well as promoting cytoskeleton stability, leading to specific neuroprotective effects in the brain (Weinreb et al., 2007). In addition, the induction of a series of hypoxia-sensitive genes regulated by HIF-1 is also affected by iron chelation. Moreover, both HIF-1 and iron-regulated protein 2 (IRP2) share a common iron-dependent proteasomal degradation pathway through the action of the key iron and O_2_ sensors prolyl hydroxylase (Erez et al., 2003; Callapina et al., 2005), whereas EGCG maintains cellular homeostasis by inhibiting both prolyl hydroxylase activity via free iron chelation and inhibiting the degradation of both HIF and IRP2. This mechanism offers a new approach for the management of NDDs (such as AD, PD, and HD) (Mandel et al., 2006).

Activation of the nuclear factor kappa-B (NF-κB)/HIF signaling axis in a hypoxic microenvironment reportedly triggers microglia activation (Liu et al., 2020; Zhang et al., 2021), which not only exacerbates the lipopolysaccharide (LPS)-induced inflammatory cascade but is also associated with the pathological process of plateau brain edema (Zhou et al., 2017). EGCG exhibits multi-targeted regulatory advantages in this segment: First, it reduces the expression of pro-inflammatory factors such as IL-1β and TNF-α by inhibiting the toll-like receptor 4 (TLR4)/NF-κB pathway (Zhong et al., 2019; Liu et al., 2016); second, it constructs a neuroprotective barrier by enhancing the BBB integrity (Wei et al., 2019; Kim et al., 2020a). Consequently, it demonstrates substantial anti-inflammatory and neuroprotective effects in AD models. Similarly, in the CoCl_2_-induced hypoxia model of BV2 microglia, EGCG achieved dual regulation of oxidative stress and inflammatory response by synergistically inhibiting the NF-κB pathway and activating the nuclear factor erythroid 2-related factor 2 (Nrf2)/heme oxygenase-1(HO-1) pathway (Kim et al., 2022). In summary, EGCG possibly prevents inflammatory responses and brain oedema in hypoxia-activated microglia through NF-κB pathway inhibition.

In addition to the NF-κB pathway, the importance of the mitogen-activated protein kinase (MAPK) signaling network in the neural injury response cannot be overlooked (Singer et al., 1999; Xia et al., 1995). As a central regulatory network in the response to nerve injury, among MAPKs, the p38 and c-Jun N-terminal kinase (JNK) pathways respond to stress stimuli (including hypoxia, free radicals, and reactive oxygen species [ROS]) (Chen et al., 2018), which promotes neurodegeneration or neuroinflammation, leading to apoptosis or cell death. Moreover, events such as hypoxia, ischemia, and infection may trigger inflammatory responses. Consequently, ischemia and hypoxia induced by long-term chronic hypoperfusion can lead to hyperstimulation of neuroinflammation, resulting in apoptosis, BBB damage, and other pathological changes. These phenomena can lead to the onset and progression of vascular cognitive impairment and dementia (VCID) (Tian et al., 2022). A study by Ashley Payne’s team offered valuable insights, as it confirmed that EGCG, by inhibiting microglial cells from aberrantly activation, effectively blocks the neuroinflammation-apoptosis vicious cycle (Payne et al., 2022), further improving the theoretical framework of its multi-pathway neuroprotective mechanism.

Furthermore, although the precise mechanism through which EGCG exerts its antioxidant effect remains unclear, its substantial capacity for free radical scavenging has been substantiated through pharmacological investigations (Guo et al., 1996; Kondo et al., 1999). Previous studies have reported that EGCG reduces lipid peroxidation damage in synaptosomes (Lin et al., 1998) and has a protective effect against neuronal damage induced by free radicals (Guo et al., 1996). This neuroprotective effect is clinically important because oxidative stress-induced lipid peroxidation not only directly leads to neuronal death (Kruman et al., 1997), but also is the central mechanism of delayed neuronal injury after transient total cerebral ischemia (Kawase et al., 1999). Moreover, animal experiments further confirmed that intraperitoneal injection of EGCG reduced neuronal damage in a dose-dependent manner in the hippocampus of Mongolian gerbils after a transient ischemic attack (Lee et al., 2000). Thus, green tea extract might be an effective preventive agent for ischemia/reperfusion (I/R) brain injury (Hong et al., 2000; Hong et al., 2001).

Complementarily, EGCG can also affect enkephalinase (NEP) activity. As a major amyloid β-peptide (Aβ) degrading enzyme in the central nervous system, NEP ameliorates AD pathology by removing Aβ (Iwata et al., 2000; Iwata et al., 2001; Nalivaeva et al., 2012). A recent meta-analysis revealed a decrease in NEP expression and activity in the cortex of older patients with AD. (Zhang et al., 2017a) I. A. Zhuravin et al. demonstrated that EGCG increased NEP mRNA levels and gene expression in human neuroblastoma NB7 cells under hypoxic conditions. Furthermore, they concluded that EGCG reverses NEP activity reduction caused by prenatal hypoxia in an animal model, and even triggered a double elevation of enzyme activity in the cortex and hippocampus (Zhuravin et al., 2019). These findings validate the neuroprotective effects of EGCG in protein homeostasis regulation, providing a new molecular basis for its application in AD therapy (Table 1).

**TABLE 1:** Pharmacological effects and molecular mechanisms of EGCG in neurological disease protection.

Cells/Model		Effects	Signaling pathways/Involved factors	Associated diseases	References
*In vivo*	*In vitro*				
-	SH-SY5Y cells	Anti-oxidative and iron chelating effects	HIF, cytoskeletal proteins, the binding protein 14-3-3 gamma↑	PD and AD	Weinreb et al. (2007)
-	-	Anti-oxidative and iron chelating effects	HIF-1, IRP2↑	PD, AD and HD	Mandel et al. (2006)
-	BV2 cells and SH-SY5Y cells	Anti-inflammatory effects	TLR4/NF-κB pathway↓	AD	Zhong et al. (2019)
SD rats	Primary human macrophages, Primary Human Neuron	Anti-inflammatory effects	TNF-α, IL-1β, and IL-6↓	MS, AD and HAD	Liu et al. (2016)
-	BV2 cells	Anti-inflammatory and anti-oxidative effects	NF-κB ↓ Nrf2/HO-1 pathway↑	Stroke and neonatal hypoxic encephalopathy	Kim et al. (2022)
Mongolian gerbils	-	Anti-oxidative effects	lipid peroxidation↓	Hippocampal neuronal damage	Lee et al. (2000)
Wistar rats	NB7 Cells	NEP activation	NEP, NEP mRNA↑	AD	Zhuravin et al. (2019)

*↑, promote, increase and positive change; ↓, inhibit, reduce and negative change. EGCG, (−)-epigallocatechin-3-gallate; SD, Sprague-Dawley; HIF, hypoxia-inducible factor; IRP2, iron-regulated protein 2; TLR4, toll-like receptor 4; NF-κB, nuclear factor kappa-B; Nrf2, nuclear factor erythroid 2-related factor 2; HO-1, heme oxygenase-1; NEP, enkephalinase; AD, Alzheimer’s disease; PD, Parkinson’s disease; HD, Huntington’s disease; MS, multiple sclerosis; HAD, HIV-associated dementia.

In summary, EGCG exerts protective effects through multiple mechanisms in various models of neurological injury, demonstrating significant potential as a novel therapeutic agent. Moreover, further research is needed in the future to enhance the physicochemical stability and oral bioavailability of EGCG.

### 3.2 CA

CA, *Centella asiatica* (L.) Urban, is a perennial herbaceous plant of the Umbelliferae family. It grows in wet or swampy areas in tropical and subtropical regions of the Northern and Southern hemispheres. Native to South Africa, India, Malaysia, Sri Lanka, Madagascar, and Australia, CA is also found in Japan and China (Gray et al., 2018). Phytochemical analyses reveal that principal bioactive constituents of CA comprise triterpenoids, polyphenols, and essential oils. The most important components of medicinal value in CA are pentacyclic triterpenic acids and the corresponding glycosides, mainly including asiatic acid (AA), madecassic acid (MA), asiaticoside (AS), and madecassoside (MS) (Zheng and Qin, 2007). This multifunctional natural product demonstrates remarkable therapeutic versatility, with established applications in wound healing (Diniz et al., 2023), dermatological disorders (Bylka et al., 2014), antimicrobial (Mudaliana, 2021), antiviral (Li et al., 2024a), and venous insufficiency (Incandela et al., 2001) as well as anxiety alleviation and cognitive enhancement (Gray et al., 2024). Its anti-inflammatory (Park et al., 2017) and antioxidant properties (Zhao et al., 2014) play a significant neuroprotective role in neuroprotection, particularly in NDDs and ischemic encephalopathy.

The neuroprotective effects of CA can be categorized into two: *in vitro* and *in vivo*. In an *in vitro* model, the presence of CA resulted in an observable increase in neurite elongation (Soumyanath et al., 2005); whereas, in an *in vivo* experiment, antioxidant capacity of CA protected the rat brain from age-related oxidative damage, confirming a significant neuroprotective effect (Subathra et al., 2005). Studies have reported that the administration of CA and its extracts exerts a neuroprotective effect on NDDs (primarily AD and PD) by selectively lowering Aβ levels (Dhanasekaran et al., 2009), inhibiting ROS production (Gray et al., 2017), ameliorating mitochondrial dysfunction (Nataraj et al., 2017), and increasing brain-derived neurotrophic factor (BDNF) levels (Nataraj et al., 2017).

Based on the scope of current research, hypoxia could accelerate the pathological progression of AD through the following mechanisms: First, hypoxia promotes the production and accumulation of Aβ, which is an important neurotoxic factor of AD (Peers et al., 2007), and is also commonly regarded as the initiator of AD; Second, hypoxia induces calcium overload by interfering with intracellular calcium homeostasis (Peers et al., 2007; Lall et al., 2019), culminating in neuronal death; moreover, hypoxia fosters the release of large amounts of ROS and proinflammatory mediators through the activation of pro-inflammatory immune cells, such as microglia (Lall et al., 2019), and this neuroinflammatory response further accelerates AD progression. Notably, CA and its active components showed multi-targeted intervention potential for several pathological processes caused by hypoxia, including inhibition of Aβ plaque deposition, regulation of calcium homeostasis imbalance, and modulation of the NF-κB/Nrf2 signaling axis (Hambali et al., 2021). Particularly, in the NF-κB pathway, the research group led by Feng Zhang discovered that mice model of acute hypoxia exhibited significantly elevated levels of NF-κB p50/p65 subunit expression and p-IκBα/IκBα ratio. This finding suggests that hypoxia activates the NF-κB pathway, thereby promoting the release of pro-inflammatory factors (Zhang et al., 2017b). From a mechanistic perspective, NF-κB pathway inhibition prevents NF-κB from dissociating from IκB, which inhibits downstream transcription of related genes and exerts neuroprotective effects. Further, the Nrf2 pathway mediates anti-inflammatory responses through its target gene HO-1 (Li et al., 2014). Notably, the Nrf2 pathway also establishes a bidirectional regulatory network through NF-κB activity inhibition (Chi et al., 2015a). In addition, the anti-inflammatory properties of CA provide neuroprotection against cerebral I/R injury. She Chen et al. found that AS significantly ameliorated I/R-induced memory impairments and inflammatory cascade responses by inhibiting microglia activation and p38 MAPK phosphorylation in the hippocampus (Chen et al., 2014).

Hypoxia-induced neurological dysfunction often involves oxidative stress, and the neuroprotective effects of CA may stem from its antioxidant properties. For example, in a rat model of acute focal middle cerebral artery occlusion (MCAO), CA significantly improved cerebral infarct size and neurological function by scavenging free radicals (Tabassum et al., 2013). Similarly, MS pretreatment exhibited antioxidant and neuroprotective effects in a GT1-7 cell model of hypoxic injury (Li et al., 2016). Notably, the mechanism of neurotrophic factors in hypoxia-induced neurodegeneration is related to oxidative stress (Sharma et al., 2019); moreover, CA regulates the expression of BDNF. Recently, the zebrafish model has provided new insights regarding the anti-hypoxic mechanism of CA. Further, Ariani et al. found that AA treatment improved head development, blood flow rate, and BDNF expression in intrauterine hypoxic zebrafish embryos (Ariani et al., 2023a), which was predicted to be associated with insulin-like growth factor-1 receptor (IGF-1R) signaling (Ariani et al., 2023b). Further studies have shown that CA also alleviates neurological dysfunction in zebrafish larvae by modulating BDNF and vesicular glutamate transporter protein 1 (VGLUT1) expressions under oxygen deprivation (OD) conditions (Ariani et al., 2024). Notably, VGLUT1 overexpression under hypoxia could trigger glutamate excitotoxicity (Daniels et al., 2011). This study also demonstrated that CA resistance to hypoxia-induced oxidative stress is also associated with VGLUT1 expression and that BDNF has a regulatory effect on VGLUT1 (Ariani et al., 2024). Furthermore, research on adult zebrafish suggests that combining CA ethanol extract and intermittent feeding ameliorates neuronal and mitochondrial damage caused by subacute hypoxia (Bindal et al., 2024). Moreover, Rajanikant et al. demonstrated that the neuroprotective effect of AA in a mouse model of permanent MCAO was associated with inhibition of mitochondrial cytochrome c release, suggesting that AA may attenuate cerebral ischemic injury by maintaining mitochondrial function (Krishnamurthy et al., 2009).

The mechanism of brain injury caused by hypoxia and ischemia is reportedly associated with neuronal apoptosis. For example, Tao Sun et al. utilized a neonatal rat cortical neuronal ischemic-hypoxic model to confirm that AS exerted an anti-apoptotic effect by regulating Bcl-2, Bax, and caspase-3 expression and inhibiting lactate dehydrogenase release (Sun et al., 2015). Similarly, a study further revealed that the neuroprotective mechanism of AS against neonatal hypoxic-ischemic encephalopathy (HIE) involved the TLR4/NF-κB/signal transducer and activator of transcription 3 (STAT3) pathway: it inhibited NF-κB phosphorylation and thus downregulated the levels of TNF-α, IL-6, and p-STAT3 by decreasing the levels of TLR4, intercellular cell adhesion molecule-1 (ICAM-1), and IL-18/IL-1β (Zhou et al., 2020). In addition, Yuan Luo et al. found that the apoptosis-inhibitory effect of MS on anti-cerebral I/R injury was also regulated by the NF-κB pathway (Luo et al., 2014). Notably, the effects of CA active ingredients on NF-κB-related pathways combine multiple anti-inflammatory, antioxidant, and anti-apoptotic effects. Similarly, by modulating the nucleotide-binding oligomerization domain containing 2 (NOD2)/MAPK/NF-κB signaling axis (including downregulation of P-ERK1/2, P-JNK, P-p38, P-65, and p-IκBα), AS achieved three major modulations of inflammation, oxidative stress, and apoptosis in MCAO rats (Zhang et al., 2020b) (Table 2).

**TABLE 2:** Pharmacological effects and molecular mechanisms of *Centella asiatica* in neurological disease protection.

Cells/Model		Effects	Signaling pathways/Involved factors	Associated diseases	References
*In vivo*	*In vitro*				
-	-	Anti-inflammatory effects	NF-κB↓, Nrf2↑	AD	Hambali et al. (2021)
ICR mice	-	Anti-inflammatory effects	p38 MAPK pathway↓	ischemic injury	Chen et al. (2014)
Wistar rats	-	Anti-oxidative effects	ROS↓, SOD↑, GSH↑	ischemic stroke	Tabassum et al. (2013)
Zebrafish embryo	-	Neuroprotective effects	BDNF↑	Perinatal HI	Ariani et al. (2023a)
	-	Neuroprotective and anti-oxidative effects	BDNF↑, VGLUT1↓	Neonatal HIE	Ariani et al. (2024)
	-	-	IGF-1R	Neonatal HIE	Ariani et al. (2023b)
Adult zebrafish	-	Anti-oxidative, anti-inflammatory, and neuroprotective attributes	AMPK↑, Nrf2↑, GSK-3β↓, MAPK↓	Ischemic stroke	Bindal et al. (2024)
C57BL/6 mice	HT-22 cells	Protecting mitochondria effects	Cyt-C↓	Cerebral ischemia	Krishnamurthy et al. (2009)
SD rats	-	Anti-apoptotic effects	TLR4/NF-κB/STAT3 pathway↓	Neonatal HIE	Zhou et al. (2020)
-	Newborn rat cortical neurons	Anti-apoptotic effects	BCL-2↑, Bax↓, caspase-3↓	Cerebral ischemia	Sun et al. (2015)
SD rats	-	Anti-oxidative, anti-inflammatory, and anti-apoptotic effects	NO↓, SOD↑, GSH↑, NF-κB↓	CIRI/stroke	Luo et al. (2014)
SD rats	PC12 cells	Anti-oxidative, anti-inflammatory, and anti-apoptotic effects	NOD2/MAPK/NF-κB pathway↓	CIRI	Zhang et al. (2020b)

^a^ ICR, institute of cancer research; SOD, superoxide dismutase; GSH, glutathione; BDNF, brain-derived neurotrophic factor; VGLUT1, vesicular glutamate transporter protein 1; IGF-1R, insulin-like growth factor-1 receptor; AMPK, AMP-activated protein kinase; Cyt-C, cytochrome c; STAT3, signal transducer and activator of transcription 3; NOD2, Nucleotide-binding oligomerization domain containing 2; HI, hypoxic ischemia; HIE, hypoxic-ischemic encephalopathy; CIRI, cerebral ischemia-reperfusion injury.

In short, CA and its extracts showed protective effects against hypoxia-induced nerve damage in many aspects. As a natural product, CA is well tolerated and has no adverse reactions (Songvut et al., 2019), and its potential is noteworthy despite the lack of relevant clinical trials based on the scope of the literature we have reviewed.

### 3.3 Ginkgolides

Ginkgo (*Ginkgo biloba*), a deciduous tree of the genus Ginkgo in the family Ginkgoaceae, is native to China and has existed for >200 million years, making it one of the most famous living fossils. Presently, dozens of compounds have been isolated from Ginkgo biloba (Meng et al., 2024). Flavonoids and terpenoids are the major bioactive compounds found in Ginkgo biloba (*G. biloba* L.). Ginkgolides are unique to Ginkgo and are the principal diterpene terpenoids in the standardized Ginkgo biloba leaf extract EGb 761, including Ginkgolide A (GA), Ginkgolide B (GB), Ginkgolide C (GC), Ginkgolide J (GJ), Ginkgolide K (GK), Ginkgolide L (GL), and Ginkgolide M (GM) (Feng et al., 2019). A distinguishing feature of all ginkgolides is the presence of a five-membered ring (Gachowska et al., 2021). The most studied ginkgolide associated with hypoxia is GB. Notably, ginkgolides possess a wide range of activities such as anti-inflammatory (Li et al., 2023; Shu et al., 2016), antioxidant (Liu et al., 2019a), anti-ischemic (Chi et al., 2015b), and neuroprotective, although they are yet to be found in any other natural plants (Liu et al., 2022; Xia and Fang, 2007; Maclennan et al., 2002). Ginkgo biloba has been recommended for medicinal use for centuries due to its high medicinal value (Hirata et al., 2019; Hassan et al., 2020). In clinical practice, ginkgo biloba extracts have been widely used to treat central nervous system disorders such as AD and cognitive deficits with little to no adverse effects (Chan et al., 2007; Peng et al., 2024; Liu et al., 2019b; Canevelli et al., 2014). This demonstrates that GB is a safe and effective adjunctive treatment option. Scientific studies have demonstrated the neuroprotective effects of ginkgolides against a variety of hypoxia-induced neuronal damage. In summary, emerging evidence reveals the superior neuroprotective potential of ginkgolides.

Zhu et al. successively investigated the neuroprotective effects of ginkgolides against neuronal injury induced by physical and chemical hypoxia. In hypoxic rat pheochromocytoma PC12 cells treated with ginkgolides, the p42/p44 MAPK pathway was activated, and HIF-1α protein expression and modification were upregulated (Li et al., 2008). To further clarify the mechanism of the neuroprotective effect of ginkgolides against cellular hypoxia injury, they investigated the neuroprotective effect of ginkgolides against chemical and physical hypoxia-induced injury in neurons and PC12 cells based on their previous results. Their findings indicated that ginkgolides significantly upregulated HIF-1α protein expression in cortical neurons, while reducing lactate dehydrogenase (LDH) release and enhancing cell viability (Zhu et al., 2007a; Zhu et al., 2007b). Therefore, the neuroprotective effects of ginkgolides against hypoxic injury might be associated with their upregulation of HIF-1α expression in hypoxic neurons.

**FIGURE 1:**
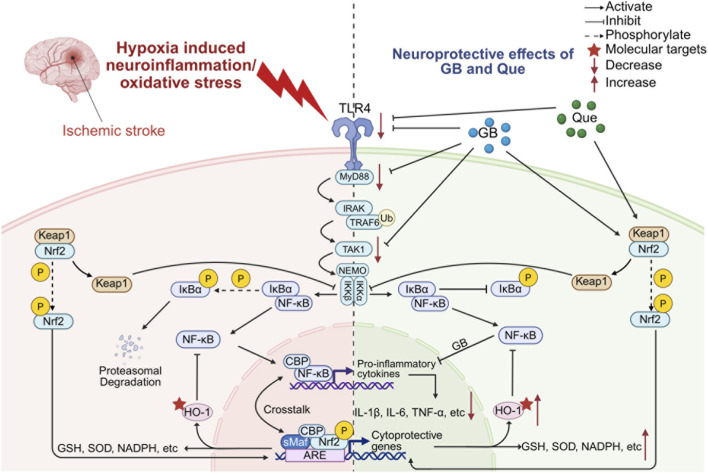
Neuroprotective effects of GB and Que on hypoxia-induced neuroinflammation and oxidative stress injury. Created in https://BioRender.com. Neuroprotective effects of GB and Que on hypoxia-induced neuroinflammation and oxidative stress injury: TLR4/MyD88/NF-κB and Nrf2/HO-1 pathways. Hypoxia activates the TLR4/MyD88/NF-κB pathway, and TLR4 recruits MyD88, activating the downstream IRAK, TRAF6, TAK1, and IKK complexes; moreover, IKKβ promotes phosphorylation of IκBα, IκBα is subsequently ubiquitinated and degraded, and NF-κB separates from it into the nucleus to initiate the transcription of inflammatory factors. GB and Que exert neuroprotective effects by inhibiting the TLR4/MyD88/NF-κB pathway, both GB and Que can reduce the expression level of TLR4, GB reduces the level of MyD88 and inhibits the phosphorylation of TAK1 and IKK, and the phosphorylation of IκBα is inhibited by both GB and Que, and GB can also inhibit the nuclear translocation of NF-κB, which culminates in the reduction of transcribed inflammatory factors. Under normal conditions, Nrf2 binds to Keap1 in the cytoplasm to form a complex and mediates Nrf2 degradation through ubiquitination and proteasomal degradation. Hypoxia-induced oxidative stress promotes the dissociation of Nrf2-Keap1 and exerts endogenous antioxidant defense effects. Keap1 negatively regulates IKKβ. Nrf2 is activated by phosphorylation and translocates into the nucleus, heterodimerizes with small Maf proteins, trans-activates the ARE genome, initiates the transcription of downstream antioxidant protein genes, such as HO-1, GSH, SOD, and NADPH. HO-1 can inhibit NF-κB translocation into the nucleus. During hypoxia, both GB and Que could activate the Nrf2/HO-1 pathway, promoting IKKβ inhibition by Keap1, and hindering the phosphorylation of IκBα, thereby inhibiting NF-κB activity. Nrf2-mediated expressions of HO-1, GSH, SOD, and NADPH are significantly increased. HO-1 also inhibits the nuclear translocation of NF-κB. Keap1, Kelch-like ECH-related protein 1; GB, ginkgolide B; Que, quercitin; TLR4, toll-like receptor; ARE, antioxidant response element; GSH, glutathione; SOD, superoxide dismutase; HO-1, heme oxygenase-1; NF-κB, nuclear factor kappa-B; Nrf2, nuclear factor erythroid 2-related factor 2.

Hypoxia reportedly reduces the activity of intracellular antioxidant systems, such as superoxide dismutase (SOD). This reduction in activity results in an imbalance between the rates of free radical production and scavenging, leading to oxidative stress (Ramanathan et al., 2005; Kleszczyński et al., 2016). Excess ROS, which are mainly produced by the mitochondria, then trigger a cascade of deleterious effects, including inflammation, neuronal damage, and irreversible brain tissue injury. In the experiments conducted by Wang et al., the activities of antioxidant defense systems, including SOD, glutathione (GSH), catalase, and other antioxidant defenses, were elevated, while malondialdehyde (MDA) concentrations were reduced in the hippocampus of rats in the GB group compared to the plateau group. These findings suggest that GB may have the potential to prevent oxidative stress (Hua et al., 2012). They therefore concluded that GB could attenuate hypoxia-induced neuronal damage in the rat hippocampus by inhibiting oxidative stress and apoptosis (Li et al., 2019). Another related study demonstrated that GB could also activate endogenous cellular antioxidant defenses by enhancing the Nrf2 signaling pathway, thereby restoring the intracerebral microenvironment in ischemic stroke and protecting brain tissues from oxidative damage (YE et al., 2022) (Figure 1).

The underlying mechanism of the neuroprotective effect of GB is not solely attributed to its free radical scavenging properties; it is believed to be more complex and involves the regulation of mitochondrial function. In a recent study, Yile Cao’s research team revealed that GB intraperitoneal injection significantly reduced neuronal loss and apoptosis in a transient MCAO model. In addition, they observed improvements in sensorimotor dysfunction, with these effects potentially being dependent on AMP-activated protein kinase (AMPK)/PTEN-induced putative kinase 1 (PINK1). *In vitro* experiments further confirmed that GB inhibited mitochondrial pathway apoptosis in an AMPK-dependent manner in hypoxia/glucose-deprived SH-SY5Y cells. In summary, GB protects mitochondrial function and reduces mitochondria-dependent neuronal apoptosis by promoting AMPK activation in neuronal cells, specifically upregulating the expression of PINK 1, which provides a novel target for intervention in ischemic stroke therapy (Cao et al., 2022).

Jian-Ming Zhou’s team has elucidated a novel anti-inflammatory mechanism of ginkgolides through the oxygen glucose deprivation and reoxygenation (OGD/R) model: significantly inhibiting the release of inflammatory factors from BV2 microglial cells through the targeted modulation of the TLRs/MyD88/NF-κB signaling pathway, and this effect demonstrates specific neuroprotective value in the treatment of ischemic stroke (Zhou et al., 2016) (Figure 1). Extensive research has demonstrated that preconditioning for transient, sublethal cerebral ischemia can enhance the brain’s resilience to subsequent ischemia and hypoxia. This phenomenon has been observed to prevent ischemic neuronal damage and exert neuroprotective effects (Liu et al., 1993; Liu et al., 1992; Kato et al., 1991; Kitagawa et al., 1991; Kirino et al., 1991). Experimental evidence showed that ginkgolide treatment induces C6 cells to produce protein expression profiles and MAPK/phosphatidylinositol 3-kinase (PI3K) pathway response patterns similar to those of hypoxic preconditioning, suggesting that it may imitate the neuroprotective programming effect of ischemic preconditioning, providing a theoretical basis for its use as a prophylaxis in ischemic-hypoxic brain injury (He et al., 2008).

In addition, several studies have reported that ginkgolides also have significant effects on extracellular amino acid levels (Thompson et al., 2012; Ahlemeyer and Krieglstein, 2003; Yang et al., 2011). Currently, emerging evidence from *in vitro* and *in vivo* experiments suggests that excitotoxicity mediated by excessive release of excitatory amino acids (such as glutamate) is an important neuropathological process in ischemic brain injury (Lai et al., 2014; Taghibiglou et al., 2009; Zhou et al., 2010; Dohmen et al., 2005). In primary cultures of neonatal rat hippocampal neurons and astrocytes, GA and GB protected neurons from glutamate excitotoxicity in a dose-dependent manner (Ahlemeyer and Krieglstein, 2003). This study also indicated that GB and GJ ameliorated apoptotic injury, which is consistent with the findings of GB inhibiting apoptosis in hippocampal neurons mentioned above (Li et al., 2019). Another related study showed that GB reduced ischemia-induced elevation of glutamate, aspartate, and glycine levels and increased extracellular γ-aminobutyric acid (GABA) levels, which means it altered the balance of excitatory and inhibitory amino acid levels in the brain and reduced excitotoxicity in the striatum of rats with MCAO, suggesting that GB ameliorated brain damage caused by cerebral ischemia-reperfusion and significantly reduced the rate of cerebral infarction in reperfused rats (Yang et al., 2011). The potential of ginkgolides to open a new pathway for stroke treatment is evidenced by the finding that it is capable of modulating the amino acid metabolic network in a multidimensional manner (Table 3).

**TABLE 3:** Pharmacological effects and molecular mechanisms of ginkgolides in neurological disease protection.

Cells/Model		Effects	Signaling pathways/Involved factors	Associated diseases	References
*In vivo*	*In vitro*				
-	PC12 cells	Antihypoxic activity	p42/p44 MAPK pathway↑, HIF-1α↑	-	Li et al. (2008)
-	Primary cortical neuron	Antihypoxic and anti-oxidative effects	HIF-1α↑, LDH↓	AD and PD	Zhu et al. (2007a), Zhu et al. (2007b)
Sprague-Dawley rats	-	Anti-oxidative effects	SOD, GSH and catalase↑ MDA↓	-	Li et al. (2019)
Sprague-Dawley rats and ICR mice	HT-22 cells	Anti-oxidative effects	Nrf2 pathway↑	Ischemic stroke	Ye et al. (2022)
Sprague-Dawley rats	SH-SY5Y cells	Anti-oxidative and anti-inflammatory effects	Bcl 2↑, PINK1↑, AMPK pathway↑	Ischemic stroke	Cao et al. (2022)
-	BV2 cells	Anti-inflammatory effects	TLRs/MyD88/NF-κB signaling pathways↓	Ischemic stroke	Zhou et al. (2016)
-	C6 cells	-	P42/p44 MAPK and PI3K/AKT/GSK-3β pathways↑	-	He et al. (2008)
-	-	Antihypoxic and anti-apoptotic and anti-excitotoxicity effects	Glu↓	AD and dementia	Ahlemeyer and Krieglstein (2003)
Sprague-Dawley rats	-	Anti-excitotoxicity	Glu, Asp, Gly↓, GABA↑	AIS	Yang et al. (2011)

^a^ LDH, lactate dehydrogenase; MDA, malondialdehyde; PINK1, PTEN induced putative kinase 1; PI3K, phosphatidylinositol 3-kinase; Glu, glutamate; Asp, aspartic acid; Gly, glycine; GABA, γ-aminobutyric acid; AIS, acute ischemic stroke.

Briefly, the diverse preclinical studies referenced above elucidate the mechanisms governing GB-induced repair of nervous system damage from a range of perspectives. Comprehensive reviews have already detailed clinical trials of GB for the treatment of AD (Pagotto et al., 2024). However, further research is needed to determine more effective administration methods, optimal dosages, and suitable drug formulations and others.

### 3.4 Que

Que is a natural plant pigment widely distributed in fruits and vegetables, particularly abundant in onions and apples (Hertog et al., 1992). Que (3,3′,4′,5,7-pentahydroxyflavone) belongs to the flavonol subclass of polyphenolic compounds, and is structured on the basis of the flavonoid C6 (ring A)-C3 (ring C)-C6 (ring B) backbone as the structural basis (Singh et al., 2021), and modified by glycosylation or methylation to form derivatives such as isoquercitrin, chrysin, rutin, and rhamnetin (Magar and Sohng, 2020). Que has a wealth of bioactivities, such as antioxidant (Hou et al., 2019), anti-inflammatory (Hou et al., 2019), anticancer (Lamson and Brignall, 2000), antimicrobial (Mlcek et al., 2016), and antiviral (Ramos et al., 2006), and has also been considered effective in offering cardiovascular protection (including antihypertensive (Kim et al., 2020b) and anti-atherosclerosis (Marunaka et al., 2017)) and neuroprotection (Luo et al., 2020). In terms of clinical applications, Que has been approved for safety in human use (Okamoto, 2005).

The neuroprotective properties of Que are manifested in several conditions, including NDDs such as AD and PD, and cerebrovascular diseases such as ischemic stroke, as well as specific neonatal neurological disorders such as neonatal hypoxic-ischemic brain damage (HIBD) and hypoxia-induced neonatal seizure (HINS). Protective effects of Que include protection of neurons from neurotoxin damage (Kaur et al., 2019), neuroinflammation inhibition (Jain et al., 2022), and improving memory and cognition (Jain et al., 2022).

Numerous studies have demonstrated the neuroprotective effect of Que against hypoxia-induced neurological injury. Hypoxia exposure induces neurodegeneration through the upregulation of HIF-1α expression. However, in an intermittent low-pressure hypoxia mouse model simulating high altitude, Que reportedly exhibits neuroprotective effects against both acute and chronic hypoxia: at the behavioral level, Que significantly improved poking and upright behavior in mice; at the molecular level, it inhibited the expression of HIF-1α and vascular endothelial growth factor (VEGF), decreased the caspase-3 activity and lowered ubiquitination levels; overall, it reduced hypoxia-induced neurodegeneration (Sarkar et al., 2012).

Furthermore, hypoxia induces oxidative stress, thereby contributing to neurodegeneration. In a model simulating high altitude, Prasad et al. discovered that by increasing the antioxidant level and free radical scavenging enzyme system, Que could reduce the level of ROS and subsequent lipid peroxidation in the hippocampus. It could also reduce the expression of caspase-3 in the hippocampus to decrease neuronal apoptosis, exerting antioxidant and anti-apoptotic effects to reverse low-pressure hypoxia-induced neurodegeneration in the rat hippocampus and enhance memory function (Prasad et al., 2013). In addition, on the effects of hypoxia on memory function, Peng Liu et al. found that Que improved acute low-pressure hypoxia-induced hippocampal mitochondrial and synaptic damage and thus memory impairment, and it was related to the peroxisome proliferator activated receptor γ coactivator-1 (PGC-1)/fibronectin type III domain-containing protein 5 (FNDC5)/BDNF pathway, which was specifically shown to modulate the silencing information regulator 1 (Sirt1), PGC-1, FNDC5, and BDNF expression (Liu et al., 2015). Generally, the above studies point to the significance of Que’s ameliorative effect on hypoxic neurological injury in high-altitude residents. In addition, evidence suggests that neuronal damage and apoptosis are closely associated with calcium dysregulation and calpain activation during hypoxia-induced oxidative stress (Aki et al., 2002). Hypoxia caused by potassium cyanide (KCN) is tissue-toxic. In an *in vitro* model of primary cultured hippocampal cells established in this manner, Que has shown strong cytoprotective and antioxidant activities, as evidenced by the maintenance of increased GSH levels and the inhibition of hypoxia-induced ROS production and intracellular Ca^2+^ influx. Reducing the activity of μ-calpain, a Ca^2+^-activated intracellular cysteine protease, has also been proposed as a potential therapeutic target for hypoxia-induced neuronal injury (Pandey et al., 2012; Pandey et al., 2016). Notably, hypoxia can lead to the expression of microRNA-122 and subsequent cell inactivation, resulting in apoptosis. Conversely, Que has been observed to enhance cell viability, promote cell proliferation and migration, and inhibit apoptosis. Rui Yan et al. discovered that Que exhibited an inhibitory effect on the damage to PC-12 cells induced by hypoxia. This effect was associated with the downregulation of miR-122 expression and the activation of AMPK and Wnt/β-catenin signaling pathways (Yan et al., 2019).

Hypoxia frequently occurs in conjunction with ischemia, and the inflammatory response and oxidative stress induced by ischemia and hypoxia can further lead to brain injury, such as ischemic stroke and neonatal HIBD, which have been the focus of more frequent research. The neuroprotective effect and mechanism of Que on ischemic stroke have been reviewed and reported in detail: Que may elicit therapeutic effects in cases of ischemic stroke by modulating oxidative stress, inflammation, apoptosis, the BBB, and ion channel regulation as well as the regulation of several signal transduction pathways, including Nrf2, NF-κB, PI3k, MAPK, Sirt1, and Wnt/β-catenin (Zhang et al., 2023) (Figure 1). Neonatal HIBD is also associated with multiple pathways, including oxidative stress, mitochondrial dysfunction, inflammatory response, and apoptosis (You et al., 2023). Several studies on the neuroprotective effect of Que on HIBD have demonstrated that it can promote remyelination, thereby improving cognitive impairment in neonatal rats caused by hypoxia and ischemia (Qu et al., 2014). It can also protect oligodendrocyte precursor cells from damage by activating the PI3K/Akt signaling pathway, downregulating caspase-3 and Bax, and upregulating Bcl-2 (Wang et al., 2011). Moreover, it inhibits the TLR4-mediated NF-κB signaling pathway to reduce brain damage, which is manifested in the partial reversal of this pathway’s representative factors TLR4, p-p65, and p-IκBα (Wu et al., 2019). Kai Le et al. further found that the attenuation of neonatal HIBD by Que was associated with inhibition of the inflammatory response mediated by the TLR4/MyD88/NF-κB signaling pathway and direct inhibition of microglia-derived oxidative stress (Le et al., 2020) (Figure 1). Regarding the TLR4/MyD88/NF-κB signaling pathway, a recent study by Zhaoyan Chen et al. elucidated its deep-rooted mechanism, which involves balancing microglia polarization and inhibiting downstream signaling by decreasing the level of Sirt1-mediated high-mobility-group protein B1 (HMGB1) acetylation (Chen et al., 2025b). In addition, more recent studies have found that Que can attenuate HIBD-induced neurodegeneration by upregulating the number of autophagosomes and the expression of nucleotide-binding domain and leucine-rich-repeat-containing family member X1 (NLRX1) in rat models of HIBD (Xu et al., 2023). Besides, Wu Yan et al. found that Que downregulated the inflammatory response via the TLR4/NF-κB pathway, showing a certain mitigating effect on HINS (Wu et al., 2022) (Table 4).

**TABLE 4:** Pharmacological effects and molecular mechanisms of Que in neurological disease protection.

Cells/Model		Effects	Signaling pathways/Involved factors	Associated diseases	References
*In vivo*	*In vitro*				
Hypoxia induced murine model	-	Anti-apoptotic effects	HIF-1a, VEGF, and caspase-3↓	High-altitude illnesses and neurodegeneration	Sarkar et al. (2012)
SD rats	-	Antioxidative and anti-apoptotic effects	SOD↑, GSH↑, caspase-3↓	Hypobaric hypoxia-induced neurodegeneration	Prasad et al. (2013)
SD rats	-	Neuroprotective effect	PGC-1/FNDC 5/BDNF pathway↑	Acute HH	Liu et al. (2015)
-	Hippocampal neurons	Anti-oxidative and neuroprotective effects	ROS↓, GSH↑	NDDs	Aki et al. (2002)
-	-	Inhibition of calpain activity	µ-calpain↓	Hypoxic injury	Pandey et al. (2016)
-	PC-12 cells	Anti-apoptotic and neuroprotective effects	miR-122↓, AMPK and Wnt/β-catenin pathway↑	Neonatal HIE	Yan et al. (2019)
-	OPCs	Anti-apoptotic effects	BCL-2↑, Bax↓, caspase-3↓, PI3K/Akt pathway↑	Neonatal HIE	Wang et al. (2011)
Male pups at postnatal day 7 (P7)	-	Anti-apoptosis, reduction of brain glial cells and anti-inflammation	TLR4/NF-κB pathway↓, BCL-2↑, Bax↓	Neonatal HIE	Wu et al. (2019)
P7 neonatal mice	Mouse BV2 microglial cells	Anti-oxidative and anti-inflammatory effects	TLR4/MyD88/NF-κB pathway↓, ROS↓, MDA↓, SOD↑, GSH↑	Neonatal HIE	Le et al. (2020)
SD rats at P7	-	Autophagy regulation	NLRX1↑, Beclin 1↑, mTOR↓, LC 3II/LC 3I↓	Neonatal HIE	Xu et al. (2023)
SD rats at P7	-	Anti-inflammation	TLR4/NF-κB pathway↓	HINS	Wu et al. (2022)

^a^ OPCs, Oligodendrocyte precursor cells; P7, postnatal day 7; VEGF, vascular endothelial growth factor; PGC-1, peroxisome proliferator activated receptor γ coactivator-1; FNDC5, fibronectin type III domain-containing protein 5; NLRX1, nucleotide-binding domain and leucine-rich-repeat-containing family member X1; mTOR, mammalian target of rapamycin; HH, hypobaric hypoxia; NDDs, neurodegenerative diseases; HINS, hypoxia-induced neonatal seizure.

To summarize, as a natural product with multiple pharmacological effects and therapeutic potential, Que has shown its neuroprotective effect in hypoxic injury. However, with the low solubility and poor bioavailability, a series of problems should be overcome to achieve clinical application.

### 3.5 BBR

BBR is a natural isoquinoline alkaloid extracted from Chinese herbs such as *Coptis chinensis and Coptis japonica*, which has a wide range of pharmacological effects (Calvani et al., 2020; Fatahian et al., 2020; Wang et al., 2021b), including good anti-inflammatory (Wu et al., 2012), anti-cancer (Sun et al., 2022), antiepileptic (Jivad et al., 2024), glycolipid metabolism (Ma et al., 2022), and neuroprotective effects (Zhang et al., 2016a). BBR has a long history in countries like China and India (Kulkarni and Dhir, 2010), and it has long played an important role in the field of Oriental medicine. BBR has a broad range of clinical applications, and various studies conducted over the years have demonstrated its beneficial effects in a variety of neurodegenerative and neuropsychiatric disorders (Yoo et al., 2006; Ji and Shen, 2011; Mohi-Ud-Din et al., 2022).

**FIGURE 2:**
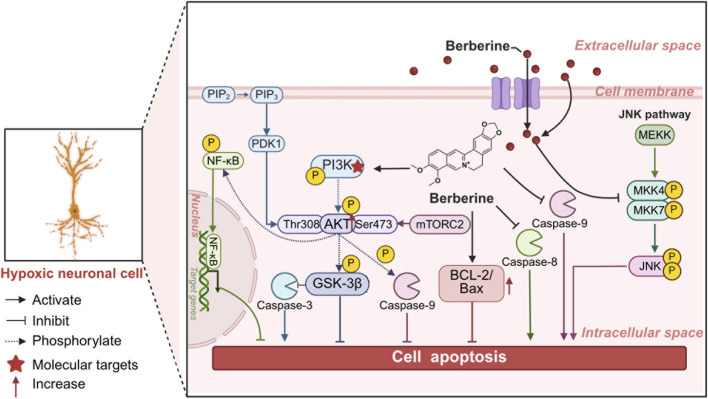
The possible mechanism of the anti-apoptotic effect of BBR. Created in https://BioRender.com. The possible mechanism of the anti-apoptotic effect of BBR: BBR enters hypoxia-induced neurons, enhances PI3K p55γ promoter activity, activates the PI3K/Akt/signaling pathway, leading to an increase in Akt and GSK3β activity, a decrease in caspase-3 activity with NF-κB and caspase-9 phosphorylation; downregulates caspase-8 and caspase-9 expression while elevating the Bcl-2/Bax ratio; and inhibits the JNK signaling pathway. NF-κB, nuclear factor kappa-B; BBR, berberine; JNK, c-Jun N-terminal kinase.

HIF-1 has been demonstrated to contribute to BBR’s capacity to target hypoxia-induced neurological impairment, a property that is consistent with its mechanism of action in other natural products. As posited by Qichun Zhang et al., the administration of BBR prior to the onset of treatment led to the activation of endogenous neuroprotective mechanisms associated with the sphingosine-1-phosphate (S1P)/HIF-1 pathway, thereby contributing to the protection of neuronal cells from the deleterious effects of hypoxia/ischemia (Zhang et al., 2016a). Notably, in addition to regulating HIF-1α, a select number of studies have demonstrated that BBR exerts anti-apoptotic and neuroprotective effects in ischemia-hypoxia models through multi-target mechanisms. In an ischemic injury model, Hu-Shan Cui et al. discovered that BBR pretreatment significantly inhibited the OGD-induced elevation of p-Bcl-2 levels in organotypic hippocampal brain slice cultures (OHSCs), and this effect was present either before or during simultaneous administration of OGD, indicating that it attenuates neuronal apoptosis by blocking Bcl-2 phosphorylation (Cui et al., 2009). Another *in vitro* study demonstrated that BBR could effectively lower intracellular ROS levels in PC12 cells, thereby inhibiting the mitochondrial apoptotic pathway, and thus exerting a neuroprotective effect against OGD-induced PC 12 cell injury (Zhou et al., 2008). Its dose-dependent neuroprotective effect is also supported by animal experiments. For example, F Benaissa et al. demonstrated that BBR has anti-stroke potential by intraperitoneally injecting BBR to significantly reduce midbrain ischemic injury and cerebral edema in rats (Benaissa et al., 2009). At the molecular level, BBR reportedly enhances PI3K p55γ promoter activity during cerebral I/R. Furthermore, BBR activates the PI3K/Akt signaling pathway, thereby inhibiting mitochondrial dysfunction. In addition, BBR reportedly reduces the cleavage of the pro-apoptotic protease, caspase-3, and thus inhibits neuronal apoptosis (Hu et al., 2012). Further, Elisa Nicoloso Simões Pires et al. revealed that BBR mediates neuroprotection by regulating the Akt/GSK3β/ERK1/2 survival/apoptosis signaling pathway while inhibiting JNK and caspase-3 activity (Simões Pires et al., 2014). Similarly, Q Zhang et al. found that BBR pretreatment significantly led to a substantial downregulation of HIF-1α, caspase-9, and caspase-3 expression while concurrently elevating the Bcl-2/Bax ratio. This study also suggests that the regulation of apoptosis in cerebral ischemic neurons by BBR may be related to the extent of cellular injury (Zhang et al., 2012). Notably, in addition to the classical apoptotic pathway, BBR also enhances neuroprotection by activating the autophagy pathway. As demonstrated by Qichun Zhang et al., it promotes autophagy by regulating autophagy markers such as LC3-II, Beclin-1, and p62. Moreover, the expression levels of caspase-8, caspase-9, poly ADP-ribose polymerase (PARP), Bcl-2/Bax and other proteins that regulate apoptosis were synergistically regulated, thereby forming an autophagy-apoptosis network (Zhang et al., 2016b). Overall, these findings suggest that BBR has the potential to mitigate the adverse effects of hypoxia/ischemia in neuronal injury (Figure 2).

The systematic analysis of the above multi-target mechanisms provides a theoretical basis for clinical applications. The study by Ke Song et al. further expands the cognitive dimension of BBR’s mechanism of action by utilizing techniques such as network pharmacology, molecular docking, and bioinformatics analysis. The team indicated that BBR may act as a neuroprotective factor by regulating the long non-coding RNA (lncRNA) H19/EGFR/JNK1/c-Jun signaling pathway in hypoxia-induced SH-SY5Y cell injury. This finding elucidates the mechanism of action and therapeutic potential of BBR in ischemic stroke (Azadi et al., 2021). Moreover, its neuroprotective effect is also reflected in the promotion of neovascularization. As demonstrated in experimental studies on animals, BBR has been observed to elicit the HIF-1β/VEGF pathway, resulting in a substantial augmentation of vascular remodeling subsequent to cerebral I/R injury (Liu et al., 2018). Overall, these studies have shown that BBR forms a three-dimensional intervention strategy against ischemic-hypoxic injury by regulating the core pathway of the hypoxic response (HIF-1α/β), remodeling the balance of cell death/survival, and promoting vascular regeneration.

Further, BBR has been demonstrated to affect not only neurons but also oligodendrocytes. Oligodendrocytes are myelin-forming glial cells of the central nervous system that are highly susceptible to ischemia-induced excitotoxic injury, which is primarily because of Ca^2+^ overload. BBR demonstrated a marked capacity to impede ischemia-induced Ca^2+^ elevation in oligodendrocytes. In addition, BBR exerted a substantial effect in attenuating excitotoxic injury and enhancing oligodendrocyte viability during hypoxia-glucose deprivation/reperfusion (Nadjafi et al., 2014a). Furthermore, an excess of intracellular Ca^2+^ induces a series of events, including NO production, which can ultimately result in cell death. The hypothesis that the blockade of NO production by BBR may also be involved in the protection of oligodendrocytes that have been injured by OGD/R is supported by available evidence (Nadjafi et al., 2014b).

In summary, BBR has potent neuroprotective properties. It is evident that BBR is a safe and effective natural product for neurological diseases. However, evidence from clinical investigations is lacking, and further clinical research should be considered in the future (Song et al., 2020).

### 3.6 Curcumin

Curcumin—also known as diferuloylmethane—is a polyphenolic natural product obtained from turmeric (Aggarwal et al., 2003), a perennial herb which originated in India and has been widely used as a spice due to its special flavor and color. It is also often found in tropical and subtropical regions such as Southeast Asia and China (Kotha and Luthria, 2019). Curcumin has a variety of biological activities, including anti-inflammatory (Khan et al., 2012), antioxidant (Kalpravidh et al., 2010), anticancer (Wright et al., 2013), antimicrobial (Wang et al., 2009), antiviral (Mazumder et al., 1995), antiangiogenic (Bhandarkar and Arbiser, 2007), antidiabetic (Chuengsamarn et al., 2012), and anti-autoimmune (Bright, 2007), and it has potential therapeutic effects on a variety of diseases, such as arthritis, diabetes, cancer, acquired immune deficiency syndrome (AIDS), and AD. Moreover, curcumin is utilized in the food industry as both a food additive and coloring agent (Aggarwal et al., 2003). Its non-toxic properties have prompted considerable research interest among scholars in relevant fields. However, the clinical application of curcumin is limited by several challenges, including its low water solubility, rapid metabolism, low bioavailability, and poor stability. As research progresses, special formulations of curcumin, such as nanoparticles, have garnered increased attention.

Recent studies have demonstrated that curcumin exerts neuroprotective effects in models of hypoxia-induced neurological injury. The underlying mechanisms of this neuroprotective response involve three primary pathways: anti-neuroinflammatory, antioxidant, and anti-apoptotic. The present study explores the potential benefits of curcumin in the treatment of NDDs (Armağan and Nazıroğlu, 2021), ischemic stroke (Zhu et al., 2022), neonatal HIE (Rocha-Ferreira et al., 2019), and other neurological disorders. A comprehensive review of the existing literature reveals the valuable role and mechanisms of curcumin.

Oxidative stress and apoptosis are important aspects of hypoxia-induced neurodegeneration. In the hypoxia-induced SH-SY5Y cell model, curcumin reportedly regulates hypoxia-induced ROS overproduction and excessive apoptosis by inhibiting the activation of transient receptor potential melastatin 2 (TRPM2) channels and intra-mitochondrial Zn^2+^ inflow (Armağan and Nazıroğlu, 2021). In addition, curcumin protects HT22 cells exposed to hypoxia from oxidative stress and endoplasmic reticulum (ER) stress, and attenuates apoptosis by modulating the expression of Prdx6 and NF-κB (Chhunchha et al., 2013). The results of the aforementioned studies demonstrated the therapeutic potential of curcumin in the treatment of hypoxia-induced NDDs such as AD and PD. Moreover, curcumin could exert neuroprotective effects against hypoxic injury by reducing neuroinflammation and increasing neurogenesis. In an intermittent hypoxia (IH) model, Bo Wang et al. found that curcumin attenuated IH-induced neuroinflammation and cerebral edema, which was achieved by inhibiting astrocyte aquaporin 4 (AQP4) expression and activating the p38 MAPK pathway (Wang et al., 2018). Based on a network pharmacology study, Yao He et al. discovered that curcumin significantly promoted neurogenesis and attenuated neurological damage by increasing Wnt/β-catenin expression (He et al., 2024a). These studies showed the therapeutic potential of curcumin for obstructive sleep apnea (OSA)-related brain injury. Notably, OSA is more common in patients with stroke or NDDs.

With regard to stroke or cerebral I/R injury, the neuroprotective effects and mechanisms of curcumin have been reported in detail in numerous reviews (Marques et al., 2022; Bavarsad et al., 2019; Fan and Lei, 2022; Du et al., 2023), including anti-inflammatory, antioxidant, and anti-apoptotic activities as well as protection of the BBB and regulation of autophagy and other pathways. These pathways involve a number of signaling pathways, such as Nrf2, PI3K/Akt/mammalian target of rapamycin (mTOR), JAK2/STAT3, and Notch. Recently, Yangyang Wang et al. applied network pharmacology and molecular dynamics to identify five key targets of curcumin against ischemic stroke, including NF-κB1, TP53, AKT1, STAT3, and TNF (Wang et al., 2023), which are mainly related to neuroinflammation, oxidative stress, and apoptosis.

Furthermore, in a study on spinal cord injury, Daverey et al. found that curcumin mediated neuroprotective effects against white matter hypoxic injury by significantly down-regulating the expression of HIF-1α, caspase-9, glial fibrillary acidic protein (GFAP), and neurofilament-H (NF-H), as well as exerting the crosstalk between NF-κB and Nrf2 signaling pathways. This finding provides a novel approach for the treatment of cerebral white matter injury (WMI) (Daverey and Agrawal, 2020).

In a brief, curcumin has important research significance for the neuroprotective properties against hypoxic nerve injury. It is worth noting that in clinical trials related to curcumin and NDDs, a clinical trial related to Amyotrophic Lateral Sclerosis (ALS) showed that nanocurcumin could improve the survival rate of ALS patients, while the results of a clinical trial related to PD did not show the efficacy of curcumin on PD, and the clinical trial of curcumin in combination with IFN β-1a for MS has not been conclusive due to high patient dropout rates and has not confirmed the neuroprotective effect of curcumin (Ghodsi et al., 2022; Petracca et al., 2021; Ahmadi et al., 2018). Although these results reveal that curcumin is well tolerated and safe (Ghodsi et al., 2022; Petracca et al., 2021; Ahmadi et al., 2018), clinical trials are susceptible to a variety of factors, and the efficacy of curcumin needs more clinical data to support it.

**FIGURE 3:**
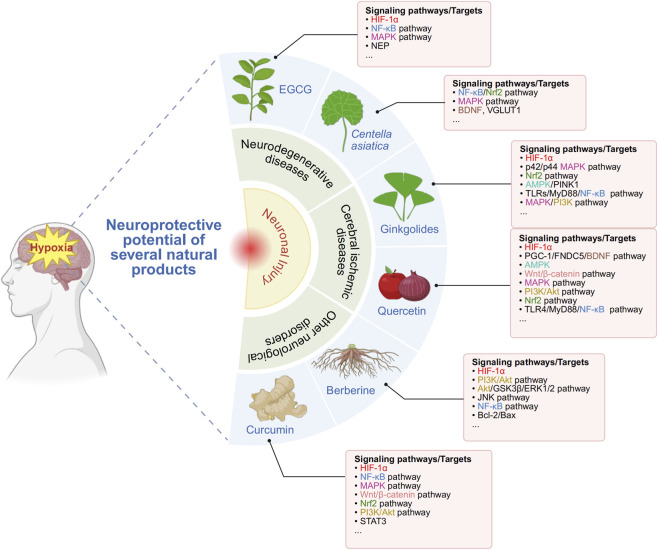
Neuroprotective potential of several natural products. Created in https://BioRender.com. Neuroprotective potential of several natural products. In this figure, Hypoxia induced neuronal injury includes neurodegenerative diseases, cerebral ischemic diseases and other neurological disorders. Natural products (EGCG, *Centella Asiatica*, Ginkgolides, Quercetin, Berberine and Curcumin) exhibit neuroprotective effects through multiple pathways/targets. We use a variety of colors to highlight the pathways/targets, where common parts are labeled with the same colors. EGCG, (−)-epigallocatechin-3-gallate; HIF-1α, hypoxia-inducible factor 1 alpha; NF-κB, nuclear factor kappa-B; MAPK, mitogen-activated protein kinase; NEP, enkephalinase; Nrf2, nuclear factor erythroid 2-related factor 2; BDNF, brain-derived neurotrophic factor; VGLUT1, vesicular glutamate transporter protein 1; AMPK, AMP-activated protein kinase; PINK1, PTEN-induced putative kinase 1; PI3K, phosphatidylinositol 3-kinase; PGC-1, peroxisome proliferator activated receptor γ coactivator-1; FNDC5, fibronectin type III domain-containing protein 5; JNK, c-Jun N-terminal kinase; STAT3, signal transducer and activator of transcription 3.

Based on the natural products discussed above, we briefly summarize the mechanisms or targets of the neuroprotective effects of these natural products on hypoxia-induced nerve injury (Figure 3).

## 4 Discussion

In this study, we reviewed the progress of recent research on the use of natural products—including EGCG, CA, ginkgolides, Que, BBR, and curcumin—for the treatment of hypoxia-induced NDDs and cerebral ischemic diseases *in vitro* and *in vivo*. Further, we summarized their effects and mechanisms of action. Hypoxia-induced NDDs and ischemic encephalopathy are associated with multiple pathways, and the common mechanisms involve neuroinflammation, oxidative stress, apoptosis, autophagy, and HIF. In addition to common mechanisms, the two types of diseases also have their own specific pathogenesis. For example, AD in NDDs often involves the deposition of Aβ plaque, while HIE is associated with glutamate over-release-mediated excitotoxicity as well as BBB damage. Targeting these pathways using natural products with anti-inflammatory, antioxidant, and anti-apoptotic properties is highly beneficial.

In neuroinflammation, hypoxia activates microglial cells in the brain to release a large number of inflammatory mediators, primarily involving the NF-κB, Nrf2, and MAPK pathways. EGCG, CA, ginkgolides, and curcumin can reduce inflammation by inhibiting the NF-κB pathway and activating the Nrf2/HO-1 pathway. Notably, NF-κB-mediated transcription can compete with Nrf2 for CREB-binding proteins (CBP), thus inhibiting Nrf2 activation; however, activating the Nrf2 pathway prevents I-κB degradation and increases HO-1 expression, thereby inhibiting NF-κB activation (Daverey and Agrawal, 2020). This bi-directional regulatory relationship is crucial for combating neuroinflammation. In addition, EGCG, CA, ginkgolides, and curcumin also attenuate hypoxia-induced inflammatory responses by inhibiting the MAPK pathway, especially in ischemic encephalopathy. Moreover, the NF-κB pathway can be activated by MAPK.

Notably, natural products counteract hypoxia-induced oxidative stress damage by scavenging free radicals (such as ROS), up-regulating the activity of antioxidant systems (including SOD and GSH), blocking Ca^2+^ inward flow, and modifying signaling pathways (including Nrf2/HO-1, NF-κB, MAPK, Peroxiredoxin 6 [Prdx6]). Among these, pathways such as NF-κB and Nrf2/HO-1 are impacted by hypoxia-induced oxidative stress when regulating the release of inflammatory factors. For example, activation of Nrf2 initiates the gene transcription of antioxidant enzymes (including HO-1 and GSH), thereby exerting an antioxidant effect.

Regarding apoptosis, natural products prevent hypoxia/ischemia-induced neuronal apoptosis primarily through the inhibition of mitochondrial apoptotic pathways, including upregulation of Bcl-2/Bax ratio, inhibition of caspase-3 activation, and mitochondrial cytochrome c (Cyt-C) release. Emerging evidence indicates that CA, Que, BBR, and curcumin have regulatory effects on the Bcl-2/Bax ratio and caspase-3, and that CA, BBR, and curcumin all inhibit the release of Cyt-C. Moreover, signaling pathways like NF-κB, MAPK, Wnt/β-catenin, PI3K/Akt, and targets like AMPK and miR-122 are also associated with anti-apoptotic effects. Specifically, Que, BBR, and curcumin can exert anti-apoptotic neuroprotective effects in the OGD model by inhibiting the activation of the PI3K/Akt pathway, while ginkgolides and Que can also counteract apoptosis in hypoxic-ischemic brain injury by activating the target AMPK.

In addition to neuroinflammation, oxidative stress, and apoptosis, hypoxia-induced neurodegenerative changes/HIE have been associated with autophagy, which is often associated with apoptosis. Autophagy maintains normal cell survival, whereas excessive autophagy leads to autophagic cell death. Autophagy-associated proteins, including LC3, Beclin-1, and p62, as well as mTOR, a core target, are involved in autophagy regulation. Notably, autophagy plays different roles in cerebral ischemia and reperfusion. Some studies have found that both Que and BBR could promote autophagy in cerebral ischemic injury (Xu et al., 2023; Zhang et al., 2016b), while curcumin inhibits autophagic activity in cerebral ischemia-reperfusion (Huang et al., 2018), thus exerting a neuroprotective effect.

In addition, HIF-1 is also involved in the process of hypoxia-induced neurodegeneration and hypoxic-ischemic brain damage; however, it has a dual role, which is related to the duration and severity of hypoxia/ischemia and other factors (Mitroshina and Vedunova, 2024; Dong et al., 2022). Generally, elevated levels of HIF-1α may act as an endogenous neuroprotective mechanism during hypoxia, which is especially significant in the early stages of hypoxia/ischemia. However, HIF-1α expression during prolonged hypoxia/ischemia may cause neuronal apoptosis or exert pro-inflammatory effects and neurotoxicity, thus exacerbating the neurodegeneration/ischemic brain injury. In addition, hypoxic preconditioning provides neuroprotective effects by activating HIF-1α (Dong et al., 2022). Pretreatment of hypoxia-induced cellular models with ginkgolides and BBR revealed upregulation of HIF-1α levels (Zhu et al., 2007a; Zhu et al., 2007b; Zhang et al., 2016a), which might be associated with endogenous neuroprotective activation of HIF-1α. However, related studies also demonstrated that reduced BBR pretreatment and Que treatment reduced HIF-1α expression (Sarkar et al., 2012; Zhang et al., 2012) and inhibited neuronal apoptosis. Moreover, EGCG inhibits the degradation of HIF-1α by prolyl hydroxylase to stabilize its structure (Mandel et al., 2006). Therefore, these natural products may exert neuroprotective effects in different hypoxia/ischemia models by the activation or inhibition of HIF-1α, and the regulatory effect is dynamically associated with the degree of hypoxia/ischemia injury. However, most studies have focused on exploring the mechanisms and validation of the therapeutic efficacy; however, the specific relationship still needs to be further elucidated by more detailed stratification studies. Further research is required to investigate how to maintain HIF-1α expression at an appropriate level to fully utilize its neuroprotective effect.

For hypoxia-induced NDDs, especially AD, reports on EGCG and CA suggest a potential therapeutic target by reducing Aβ plaque deposition. In addition, dopaminergic neurodegeneration of PD may be linked to hypoxia-induced oxidative stress and neuronal apoptosis, with natural products such as CA, GB and curcumin also exerting potential protective effects (He et al., 2024b; Yu et al., 2021; Patel et al., 2022). Whereas for hypoxic-ischemic brain damage with excessive glutamate release-mediated excitotoxicity and impaired BBB, CA and ginkgolides show potential for modulation of glutamate; EGCG, Que, and curcumin are essential in protecting the BBB.

Notably, in addition to the six natural products reviewed in this study, several other natural products also have neuroprotective effects against cerebral hypoxia/ischemia injury. Resveratrol, another polyphenol, exhibits anti-inflammatory effects on microglia by inhibiting the NF-κB/ERK/JNK MAPK signaling pathway, thereby counteracting the neurotoxicity caused by hypoxia (Zhang et al., 2015), demonstrating neuroprotective effects against hypoxia-induced oxidative stress and apoptosis (Auti et al., 2022). In addition, most of the relevant research is now centered on the individual actions of natural products, with limited research on compounded preparations. Among these, Trans-Himalayan phytococktail (PC) is a preparation composed of sea buckthorn, mountain almond, and rhodiola rosea—natural plants from the Himalayan region. PC can prevent low-pressure hypoxia-induced hippocampal neurodegeneration and neuronal apoptosis by activating the PI3K/AKT signaling pathway (Dhar et al., 2019). The neuroprotective effects, mechanisms of action, and clinical applications of these natural products or compounded preparations are equally valuable research areas.

At the disease level, hypoxia is closely associated with neurodegeneration, and as such, the development of prevention and treatment strategies for NDDs based on hypoxia-mediated pathological mechanisms is of great clinical value. However, compared with ischemic diseases, studies on the association between NDDs and hypoxia remain limited. Moreover, studies have reported that cerebral hypoperfusion and hypoxia in patients with ischemic stroke interact with the pathological process of NDDs (Nucera and Hachinski, 2018). Among ischemic diseases, in addition to stroke, which is prevalent in older adults, age-specific diseases like neonatal HIE are also of concern. Furthermore, individuals who are chronically exposed to low-pressure hypoxic environments at high altitudes, or who climb mountains, travel to high plateaus, or fly airplanes, are potentially at risk for cognitive decline and neurodegeneration (Prasad et al., 2013; Liu et al., 2023; Kushwah et al., 2018). Natural products such as EGCG, ginkgolides, and Que have significant neuroprotective effects on neuronal damage associated with hypoxia at high plateaus, providing a potential strategy for developing targeted interventions.

However, relevant studies still have some potential limitations. For instance, the precise mechanisms underlying antioxidant activity of EGCG, as well as the ability of CA to combat hypoxia-induced neuroinflammation, remain unclear. Moreover, effective clinical trials are still lacking, with a large number of studies still focusing on *in vitro* and/or *in vivo* experiments. Despite sufficient preclinical evidence, the clinical application of natural products still faces challenges such as low bioavailability, poor BBB penetration, and poor stability, emphasizing the need for further research. The majority of the discussed natural products exhibit pharmacokinetic (PK) limitations, such as poor water solubility, fast metabolism and limited route of administration, which should be addressed (Peng et al., 2025; Manach et al., 2005; Lehoczki et al., 2025). Furthermore, several formulation strategies have been developed to overcome these PK issues. Some natural products, such as Que and curcumin, have been proposed to improve water solubility and bioavailability with targeted nanodelivery technology and other methods including changing the intrinsic properties by designing and synthesizing new derivatives, as detailed in the review of quercetin drug design and development by Zhang et al. (2023), Yavarpour-Bali et al. (2019), Alizadeh et al. (2022). Moreover, there is a paucity of systematic comparisons of the synergistic effects and dose-response relationships between different active ingredients.

Regarding future perspectives, we cannot ignore the continuous increase in the application of artificial intelligence (AI) in recent years, as it has contributed largely to data analysis, disease modeling, drug target identification, disease prognosis prediction, and other aspects. The construction of PD progression trajectory models with the help of AI algorithms (Birkenbihl et al., 2023), development of AD neuroprotective agents by combining deep learning with pharmacological studies (Li et al., 2024b), and application of AI to optimize acute stroke treatment (Liaw and Liebeskind, 2020) suggest that the integration of machine learning, such as AI, could be indispensable in the study of the mechanisms of action of natural products, and how they mitigate hypoxic neurological damage as well as for the intervention and treatment of hypoxic neurological disorders. In addition, many natural products still face the problem of low bioavailability and lack of detailed clinical data. In the future, more methods to improve bioavailability need to be developed and more clinical trials need to be conducted.

## 5 Conclusion

Among the neurological diseases, NDDs and cerebral ischemic diseases are particularly significant. They exhibit notable differences and potential correlation in terms of pathogenic mechanisms, disease progression, and clinical interventions. Recently, various forms of hypoxia (including acute, chronic, and intermittent hypoxia) have gradually highlighted their roles in the pathogenesis of NDDs and cerebral ischemic diseases, greatly affecting both disease progression and regression; however, targeted clinical interventions or treatments remain limited. Therefore, we reviewed several natural products with significant neuroprotective effects against hypoxia-induced neurological injury, including EGCG, ginkgolides, CA, Que, BBR, and curcumin, with common features including anti-inflammatory, anti-oxidative stress, and anti-apoptosis, as well as exerting neuroprotective effects through multiple pathways and targets. Natural products have great therapeutic potential and promising application prospects for hypoxia-induced NDDs, ischemic encephalopathies, and other diseases. Furthermore, compared with current medications for the treatment of neurological diseases, they possess distinctive advantages of fewer adverse effects and higher safety. As technology and research progress, the detailed mechanisms of action of these natural products will be supplemented and improved, challenges like low bioavailability will be solved, and pertinent clinical trials will be conducted. By systematically sorting out the existing research results, we hope that this review will provide novel approaches for the development of natural drug-based preventive and therapeutic strategies for hypoxia-induced NDDs, stroke, and other diseases.
